# Theoretical Modeling for the Thermal Stability of Solid Targets in a Positron-Driven Muon Collider

**DOI:** 10.1007/s10765-021-02913-x

**Published:** 2021-09-03

**Authors:** Gianmario Cesarini, Mario Antonelli, Fabio Anulli, Matteo Bauce, Maria Enrica Biagini, Oscar R. Blanco-García, Manuela Boscolo, Fausto Casaburo, Gianluca Cavoto, Andrea Ciarma, Francesco Collamati, Cyril Daout, Roberto Li Voti, Alessandro Variola

**Affiliations:** 1 I.N.F.N. Sezione di Roma, Piazzale Aldo Moro 2, 00185 Rome, Italy; 2 I.N.F.N. Laboratori Nazionali di Frascati, Via Enrico Fermi 40, 00044 Frascati, Italy; 3 Dipartimento di Scienze di Base ed Applicate per l’Ingegneria, Sapienza Università di Roma, Via Antonio Scarpa 16, 00161 Rome, Italy; 4 Dipartimento di Fisica, Sapienza Università di Roma, Piazzale Aldo Moro 2, 00185 Rome, Italy

**Keywords:** Muon collider, Nonlinear heat transfer, Solid targets, Thermal stress, Thermomechanics

## Abstract

A future multi-TeV muon collider requires new ideas to tackle the problems of muon production, accumulation and acceleration. In the Low EMittance Muon Accelerator concept a 45 GeV positron beam, stored in an accumulation ring with high energy acceptance and low angular divergence, is extracted and driven to a target system in order to produce muon pairs near the kinematic threshold. However, this scheme requires an intensity of the impinging positron beam so high that the energy dissipation and the target maintenance are crucial aspects to be investigated. Both peak temperature rises and thermomechanical shocks are related to the beam spot size at the target for a given material: these aspects are setting a lower bound on the beam spot size itself. The purpose of this paper is to provide a fully theoretical approach to predict the temperature increase, the thermal gradients, and the induced thermomechanical stress on targets, generated by a sequence of 45 GeV positron bunches. A case study is here presented for Beryllium and Graphite targets. We first discuss the Monte Carlo simulations to evaluate the heat deposited on the targets after a single bunch of 3 × 10^11^ positrons for different beam sizes. Then a theoretical model is developed to simulate the temperature increase of the targets subjected to very fast sequences of positron pulses, over different timescales, from ps regime to hundreds of seconds. Finally a simple approach is provided to estimate the induced thermomechanical stresses in the target, together with simple criteria to be fulfilled (i.e., Christensen safety factor) to prevent the crack formation mechanism.

## Introduction

The particle physics community is currently in the process of identifying next generation particle colliders to advance in the study of fundamental interactions. Among other options under investigation, the possible realization of a muon ($\mu$) collider is providing a challenging though very exciting opportunity, due to its invaluable sensitivity to investigate new realms of physics [1–3]. The production of $\mu$ beams is typically achieved via the interaction of protons with an energy of a few GeV with stationary targets [4–6]. In this classical production scheme, however, the produced muon beam is characterized by a significant angular divergence, being the result of secondary particles decay. As a consequence, a beam-cooling procedure, able to reduce the 6D emittance by several order of magnitudes, is needed in order to reach the required high luminosity at the final particle colliding stage.

In this paper, instead, an alternative muon production scheme is considered, leading directly to high quality muon beams, based on electron–positron collisions at a centre-of-mass energy just above the ${\mu }^{+}{\mu }^{-}$ production threshold [7, 8]. One of the main drawbacks of this scheme is, however, the requirement for a very intense positron beam, given the very low cross section of the ${e}^{+}{e}^{-}\to {\mu }^{+}{\mu }^{-}$ process. A possible example of such a $\mu$ production approach is constituted by a ~ 45 GeV positron beam colliding on a stationary electron target. The key feature of the muon beam production via positron-on-target is the high level of collimation of the produced particles, corresponding to a small spread in particle transverse momentum distribution, with no need—in principle—of a subsequent cooling stage. The produced muons have an average momentum of about 22 GeV and, thanks to their relativistic boost, an average lifetime of almost 460 µs in the laboratory reference system, significantly increased with respect to the proper muon lifetime (2.2 µs). As far as the muon bunch intensity is concerned, the number $dn$ of ${\mu }^{+}{\mu }^{-}$ pairs produced per positron bunch on a given target with thickness *dl* is given by the equation:

1 $$dn\left({\mu }^{+}{\mu }^{-}\right)={n}^{+}{\rho }^{-}\sigma \left({\mu }^{+}{\mu }^{-}\right)dl$$

where *n*^+^ is the number of *e*^+^ in the bunch, ${\rho }^{-}$ is the electron density in the target medium, and $\sigma \left({\mu }^{+}{\mu }^{-}\right)$ is the muon pair production cross section for the process ${e}^{+}{e}^{-}\to {\mu }^{+}{\mu }^{-}$. Muon pairs can be produced when the energy of the center of mass $\sqrt{s}$ is above the kinematic production threshold of ~ 0.21 GeV. In a range close to the threshold value for $\sqrt{s}$ the maximum of $\sigma \left({\mu }^{+}{\mu }^{-}\right)$ is about 1 µb. A campaign of measurements to study the features of this production scheme has been recently performed at CERN, by means of positron beams of energies between 45 and 49 GeV colliding on a Beryllium (Be) or Carbon (C) target [9, 10]. Further investigations are planned to precisely measure the production cross section and the emittance of the produced muon beams.

To achieve a reasonable $\mu$ production efficiency and hence the desired $\mu$ beam intensities, this configuration requires a target with very high electron density. Such high-density values can be obtained either in a liquid or a solid target or, possibly, in more exotic solutions like crystals [11]. In order to preserve the quality of the produced muons, the target density should be sufficiently low to reduce the effect of multiple Coulomb scattering of the particles in the material.

Relatively low-Z materials such as Be and C are nowadays in use or under study for collimators in high energy physics accelerators also given the stringent vacuum requirements in such complexes, not easy to fulfil with liquid targets. Recently developed carbon-based materials with excellent thermo-mechanical properties are, for example, under consideration for the collimators of the Large Hadron Collider (LHC) upgrade at CERN. A 7.5 µs long beam pulse made of 288 bunches with 1.2 × 10^11^ protons per bunch, which is the full LHC injection batch extracted from Super Proton Synchrotron (SPS), has been used to test both C-based [12] and Be-based [13] targets with maximal temperatures reaching 1000 °C. These studies demonstrated that such targets can sustain the power load of the SPS pulse for a beam spot size down to 0.3 mm^2^. Since for thin and light-material targets the energy deposition of high energy protons and positrons is approximately the same, these studies suggest a 10^19^ e^+^/(s⋅mm^2^) intensity as achievable.

This article aims at a systematic study of thermo-mechanical stress of the target in these unique conditions. Detailed procedures, reliable methods and accurate models have been developed to be effectively applied in the estimation of the damage occurring during beam-target collisions.

**Fig. 1:**
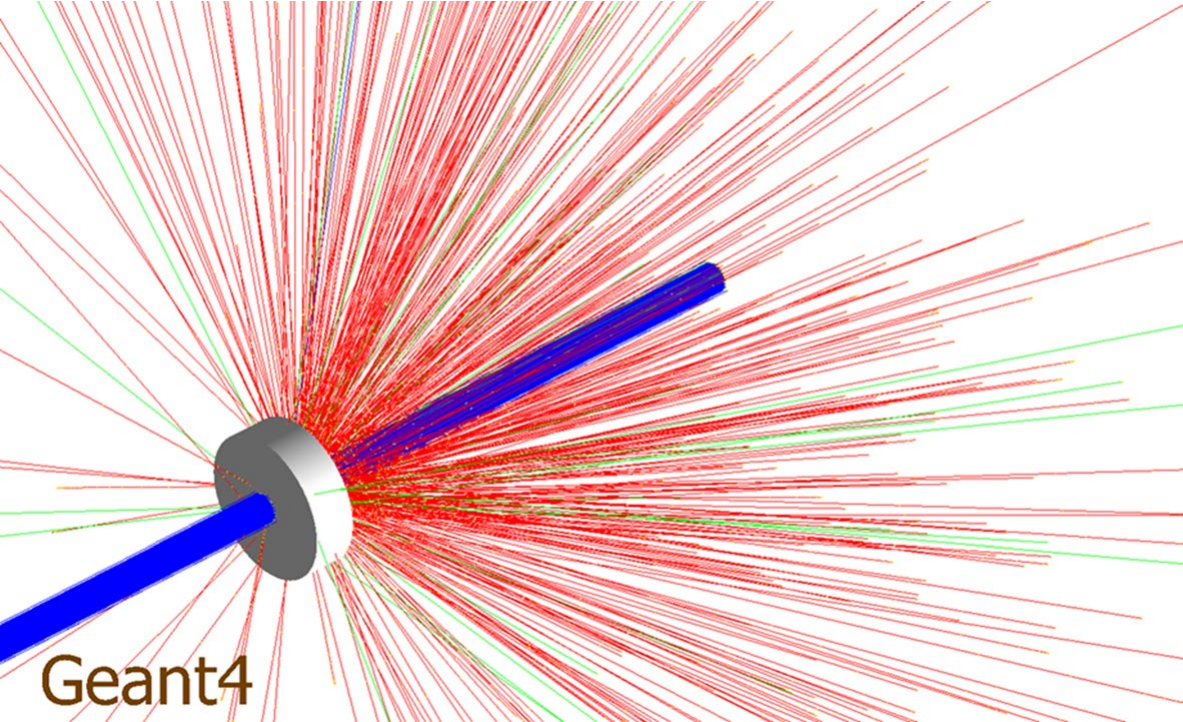
Visualization, obtained by Geant4, of a primary 45 GeV positron beam (blue, coming from the left) hitting a 3 mm beryllium target and producing secondary particles (red and green lines)

The time pattern of the incoming positron beam and of the solid target considered as input for the thermo-mechanical analysis is described in Sect. 2, together with the description of the simulation used to obtain the energy deposition map, based on the FLUKA particle transport and interaction [14, 15] and Geant4 (Fig. 1) [16] Monte Carlo codes. These are considered as input for the thermo-mechanical analyses described in Sects. 3 and 4, where the details of space–time temperature field evolution and thermal stress analysis are reported, respectively. In Sect. 5 the results of this study are discussed.

## Beam and Targets

In this Section we describe the setup that we considered as input in the theoretical calculation developed to assess the thermomechanical stress of the targets. The peculiar configuration follows the requirements for the realisation of a positron-driven muon collider, a quite challenging and unique condition. Section 2.1 describes the beam timing structure considered in the subsequent studies, Sect. 2.2 reports the parameters of the solid targets that have been examined, while Sect. 2.3 eventually evaluates the heat density deposited in the targets, which produces the temperature increase and the thermomechanical stress in the whole volume.

### Beam Timing Structure

**Fig. 2:**
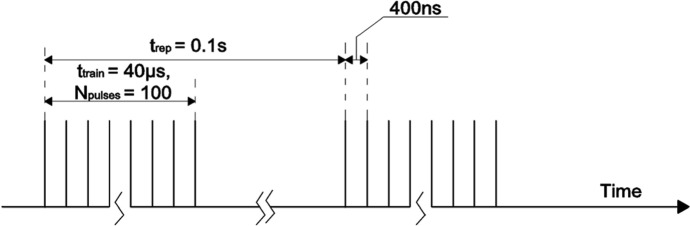
A schematic representation of the bunch structure considered in this study. The beam is composed of bunches (pulses) 10 ps long, separated from each other by 400 ns and gathered in trains of 100 bunches. Each train is therefore 40 μs long and separated from the following one by 0.1 s

To achieve a reasonable muon production rate [7] we consider a pulsed positron beam of 45 GeV, as depicted schematically in Fig. 2.

The beam consists in a sequence of trains of 100 positron bunches, each bunch containing 3 × 10^11^ positrons. The positron arrival time distribution on the target is assumed to be uniform within the bunch length of 10 ps. Within a single train, bunches are spaced in time by 400 ns, resulting in a total time span of the train of 40 μs. We consider a repetition rate of trains colliding on the target system of 10 Hz, so that two consecutive trains are separated in time by *t*_*rep*_ = 0.1 s. Considering this type of beam, the average rate of positrons impinging on the target is ~ 10^14^ e^+^ s^−1^, but the peculiar timing structure leads to a peak rate of ~ 10^18^ e^+^ s^−1^ when each train is crossing the target. The considered positron beam has a Gaussian radial density profile, with a standard deviation that has been varied in the 150–1000 μm range in this study. Hereinafter, we refer to this beam Gaussian profile standard deviation as *beam spot*. The relevant parameters of the considered pulsed beam structure are summarized in Table 1.

**Table 1:** Reference parameters of the spatial and temporal structures of the pulsed positron beam

Symbol	Description	Reference value
***a***	Gaussian beam spot size	150–1000 μm
***τ***	Bunch duration	10 ps
*N*_part_	Number of positrons per bunch	3 × 10^11^
*N*_pulses_	Number of consecutive bunches	100
*t*_pulse_	Time interval between bunches	400 ns
*t*_train_	Total time length of a train	40 μs
*t*_rep_	Time interval between two trains of bunches	0.1 s

### Target Materials

This study considers solid targets made of Beryllium [17–22] and Carbon (Pyrolytic Graphite) [23, 24], which have been found to be suitable candidates to sustain the demanding conditions of the muon production scheme described in Sect. 1. These materials have relatively low atomic number *Z* = 4 and *Z* = 6 and electron density of about 5 × 10^23^ e^−^⋅cm^−3^ and 7 × 10^23^ e^−^⋅cm^−3^ for Beryllium and Carbon (Pyrolytic Graphite), respectively. Given the expected power load due to the impinging positron beam and the consequent temperature increase, it is mandatory to consider the explicit dependence of the thermal and mechanical parameters of these materials on temperature [25]. These dependencies and their non-linearity play a decisive role in the assessment of thermo-mechanical stresses, cooling time and dissipation modes of the energy deposited on the target.

**Fig. 3:**
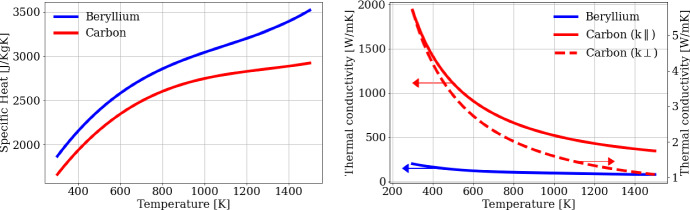
Thermal parameters of Beryllium and Carbon (Pyrolytic Graphite) as a function of the temperature in the range 300–1500 K. The solid and dashed red lines on (right) represent the parallel and orthogonal components of the Carbon thermal conductivity, and the arrows indicate the reference axis

For the modeling of the target thermal evolution, we consider the relations of the specific heat and of the thermal conductivity directly to temperature for Beryllium [26–31] and Carbon [32–34]. The dependences on the temperature *T* of the thermal conductivity *k* (*k*_*∥*_, *k*_*⊥*_ representing the parallel and orthogonal component of *k* with respect to the sample surface), and of the specific heat ${c}_{p}$ are reported in Fig. 3.

**Fig. 4:**
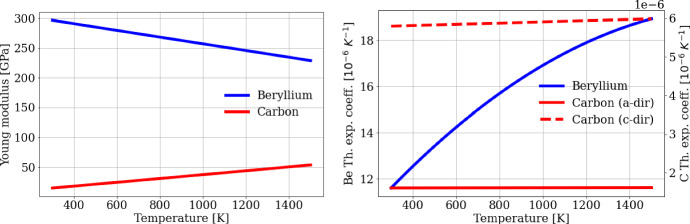
Structural parameters of Beryllium and Carbon (Pyrolytic Graphite) as a function of the temperature in the range 300–1500 K. The solid and dashed red lines (right panel) represent the thermal expansion coefficient along the crystalline directions a and c of the Carbon

In the same way, for Beryllium and Carbon (Pyrolytic Graphite), we consider the dependence on the temperature of the structural properties of these materials, which are strongly affecting the maintenance of the target’s operativeness. Figure 4 shows the variation of the Young modulus and of the thermal expansion coefficient in the same temperature range.

### Energy Deposition Density

When a positron from the beam interacts with the target material, different processes occur, leading to different energy loss mechanisms [35]: inelastic collisions with the orbital electrons (*ionization*), inelastic collisions with the atom nuclei (*bremsstrahlung*), and elastic collisions with the atom nuclei (*Rutherford scattering*) to quote the most frequent.^1^ In the energy range of the considered positron beam, bremsstrahlung is the dominant process through which positrons are losing energy when crossing the target. In this process, the positron emits a photon, with a large energy spectrum, up to the kinematical threshold. Low energy photons, in the range of O(10–100 keV), are usually absorbed through atom photo-ionization. Photons emitted with energy larger than 1 MeV can instead produce multiple secondary particles in the material in the form of an electromagnetic cascade. When all the produced secondary particles reach an energy below a critical threshold, the multiplication process stops, and the initial emitted radiation can be considered as fully absorbed by the material. Eventually all these processes induce a thermal energy load on the target material.

In this paper, we evaluate the energy deposition in two solid targets made of Beryllium and Carbon, respectively. The targets have a disk shape^2^ with radius *R* = 5 cm, and a thickness of a few millimeters (3 mm for Beryllium and 1 mm for Carbon). The different thickness of the considered targets is chosen so that the amount of energy deposited by a positron in each of the two targets due to *bremsstrahlung*^3^ is similar, with a value of about 1 MeV. Given Eq. 1, this thickness corresponds to about one muon pair produced every million of 45 GeV positrons impinging on the target. The accurate evaluation of the energy deposited in the target by the positron beam has been obtained with FLUKA and Geant4.

**Fig. 5:**
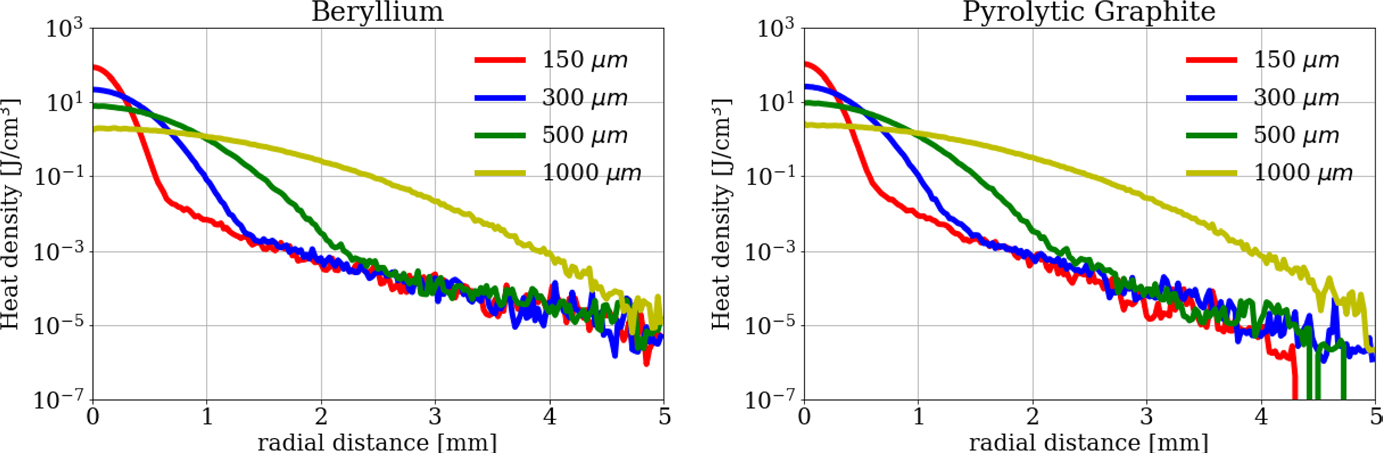
Deposited Energy density from a single bunch of 3 × 10^11^ positrons on (left) a 3 mm thick Beryllium target and (right) a 1 mm thick Graphite target for different beam spot sizes

Figure 5 shows the heat density deposited by a single bunch of *N*_e+_  = 3 × 10^11^ positrons crossing the target, as a function of the radial distance from the center, for Beryllium (a) and Graphite (b), respectively. Different values of the beam spot size (*a*) have been considered in the range 150–1000 µm: the curves corresponding to *a* = 150  µm, 300  µm, 500  µm, 1000 µm are shown in Fig. 5. Similar heat density distribution in the Beryllium and Carbon targets for the considered *a* values are found, even though the heat density level in Carbon is on average 1.4 times larger than the corresponding one in Beryllium.

The positron beam pulse is producing in the target a heat density profile, found to be approximately Gaussian with the same radial size as that of the beam, with a peak value of *q*_max_ = 20.8 J⋅cm^-^^3^ in the case of 3 mm thick Beryllium disk and *q*_*max*_ = 28.1 J⋅cm^-^^3^ in case of 1 mm thick Carbon disk, for a spot size *a* = 300 µm. Small differences are present outside the bulk of the beam spot, typically with a deposited energy of 2–3 orders of magnitude smaller than the peak one. Assuming a constant number of impinging positrons, we verify that an increase of *a* results in a decrease of the heat density peak value as 1/*a*^2^, as expected.

**Fig. 6:**
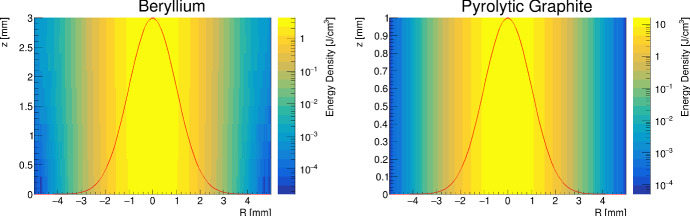
Deposited energy density, obtained by Geant4, from a single bunch of 3 × 10^11^ positrons and a beam spot size of 1000 μm on (left) a 3 mm thick Beryllium target and (right) a 1 mm thick Graphite target; the Gaussian function (red line) has been drawn to underline the energy density profile (colour figure online)

The variation of the deposited energy density as a function of the depth *z* is shown in Fig. 6 for a single beam spot size. Given the penetrating nature of beam positrons and radiated photons, a negligible dependence along the *z* direction is shown.

## Space–Time Temperature Field

Starting from the Monte Carlo simulations of the heat density described in Sect. 2.3, we introduce the theoretical approach to study the evolution of the temperature profile in the target at different time scales from the picosecond regime to hundreds of seconds. Section 3.1 describes the theoretical model to simulate the heat diffusion process inside the material, and the heat exchange with the environment via infrared radiation, so to evaluate the space–time temperature field. The theoretical results of the numerical simulations for both Beryllium and Graphite targets are discussed in Sect. 3.2, comparing the different performances and the thermal stability of the materials.

### Theoretical Model

In this Section we discuss the temperature field induced by a positron beam impinging on a solid target. The space–time distribution of the temperature field has been calculated from the FLUKA and Geant4 simulations [14–16] of the heat density deposited in the target, and by solving the Fourier heat transfer equation with the appropriate boundary conditions.

A dedicated algorithm based on Finite-Difference Time Domain method (FDTD) has been implemented to evaluate, on different time scales, the heat diffusion processes occurring in the target when hit by multiple trains of bunches. As said, the space–time distribution of the induced thermal field should fulfill the Fourier heat diffusion equation.

2 $$\nabla \cdot \left(k\cdot \nabla T\right)+w=\rho {c}_{p}\frac{\partial T}{\partial t}$$

where *ρ* is the density and *w* the deposited power density. Equation 2 should be completed with the boundary conditions at each border of the target, by imposing the heat exchange with the environment via heat radiation only.^4^

The problem has a clear axial symmetry due to the Gaussian shape of the positron beam, and due to the cylindrical shape of the target. Therefore the FDTD algorithm [37, 38] can be implemented in a 2D space, i.e., in the *r, z* domain, with a clear reduction of the complexity [39–43], allowing to study the heat diffusion along both the radial (i.e., the dominant contribution) and axial directions.

**Fig. 7:**
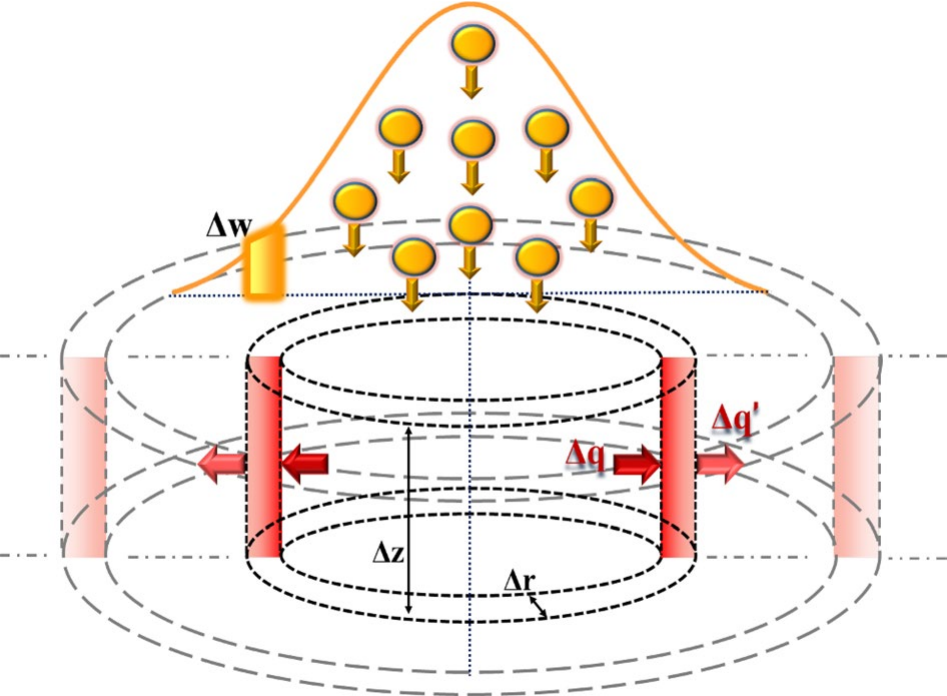
Sketch of the positron Gaussian beam and of target discretization in (Δ*r*, Δ*z*) cylindrical elements (voxels); **Δw** represents the amount of heat deposited per unit time from the positron beam in a single voxel, **Δq** the heat flowing in the voxel and **Δq’** the heat flowing out of the voxel

The cylindrical target is discretized in a number of small cylindrical shells (voxels) of volume *ΔV = 2πrΔrΔz* (see Fig. 7), corresponding to the 2π revolution of the area Δ*r⋅*Δ*z* in the 2D space (see Appendix B). In each voxel, temperature is assumed homogeneous. The thermal conductivity, specific heat and thermal diffusivity (*D* = *k/ρc*_*p*_) depend on temperature (see Sect. 2.2), and therefore should be recalculated in each voxel, for each integration time Δ*t*.

The heat exchange among neighboring voxels is regulated by discretizing Eq. 2, which should be written for each voxel. The system of coupled equations is solved by the FDTD algorithm, obtaining the temperature of each voxel for each time interval Δ*t*. It has to be noted that the target is considered to be initially in equilibrium at room temperature *T*_*0*_ = *T*_*room*_ = 300 K.

The choice of Δ*t* is fundamental to guarantee the convergence of the numerical heat transfer model. The convergence criteria are satisfied if the Fourier number, *Fo*, and consequently Δ*t*, fulfill the stability condition

3 $$Fo=\frac{D\Delta t}{{L}^{2}}\le \frac{1}{2} \Rightarrow \Delta t<\frac{{\text{min}}\left({\Delta r}^{2},{\Delta z}^{2}\right)}{2{D}_{\text{max}}}$$

where *D* is the target thermal diffusivity, and *D*_max_ is its maximum value reached during the temperature evolution.

Further technical details concerning the heat transfer numerical model are reported in Appendix B.

However, in order to evaluate the temperature increase of the target in the long timescale of hundreds of seconds, the FDTD algorithm becomes inappropriate due to the huge computational time required to follow the temperature increase of every voxel to be updated with the considered temporal step Δ*t* (~ μs). An alternative approach, which guarantees a fast solution, is given by a simple model based on the energy balance between the heat deposited in the target by the pulsed positron beam and the energy emitted from the target via infrared radiation [44–49], according to the expression

4 $${P}_{\text{cw}}-\varepsilon \sigma \left({T}^{4}-{T}_{\text{room}}^{4}\right)S=m{c}_{p}\frac{\partial T}{\partial t}$$

where *T* is the average target temperature, *P*_*cw*_ = *E*_*d*_*/t*_rep_ is the equivalent “*continuous wave power*” deposited on the target as if the whole energy *E*_d_ was continuously deposited during the whole period *t*_rep_ between two subsequent trains of bunches, *m* is the mass of the target, *S* is the total surface of the target, ε is the sample emissivity, *T*_room_ is the temperature of the environment. The nonlinear ordinary differential Eq. 4 can be numerically solved iteratively by the Runge–Kutta method, eventually finding the time evolution of the target temperature *T*(t).

It is worth noting that in the long timescale the sample temperature tends to reach an asymptotic steady state temperature *T*_*SS*_, which can be estimated by applying the stationary condition $\frac{\partial T}{\partial t}=0$ to Eq. 4, so to obtain

5 $${T}_{SS} = \sqrt[4]{{T}_{\text{room}}^{4}+\frac{{E}_{d}/{T}_{rep}}{\varepsilon \cdot \sigma \cdot S}}=\sqrt[4]{{T}_{\text{room}}^{4}+\frac{{a}^{2}\cdot L}{R\cdot \left(R+L\right)}\frac{{N}_{\text{pulses}}\cdot {q}_{\text{max}}}{\varepsilon \cdot \sigma \cdot {T}_{\text{rep}}}}$$

where the total surface has been put as *S* = *2*π*∙R(R* + *L)*, being *R* and *L* the radius and the thickness of the cylindrical target, while the heat deposited by one train has been written as *E*_*d*_ = (*2*π*a*^*2*^*L)∙N*_pulses_*∙q*_max,_ being *N*_pulses_ the number of bunches per train, and *q*_max_ the maximum heat density in the beam center deposited by a single pulse of 3 × 10^11^ positrons, as calculated by the FLUKA and Geant4 numerical simulations (see Fig. 5). As a final remark, we underline that this approach allows a quick evaluation of the target average working temperature dynamics, but loosing information on the internal thermal gradients, and consequently on the induced thermomechanical stresses, as will be discussed in Sect. 4.

### Numerical Simulations and Discussion

**Fig. 8:**
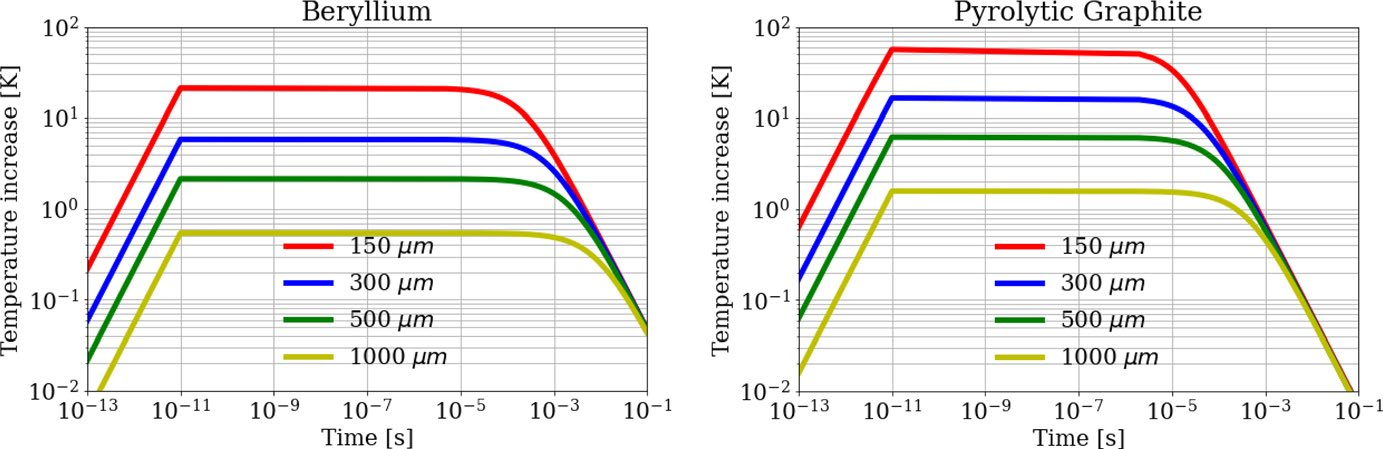
Temperature rise in the position corresponding to the beam center (*r* = 0), as a function of time, for a single bunch of 3 × 10^11^ positrons impinging on the (left) Beryllium and (right) Carbon target for different beam spot sizes

**Fig. 9:**
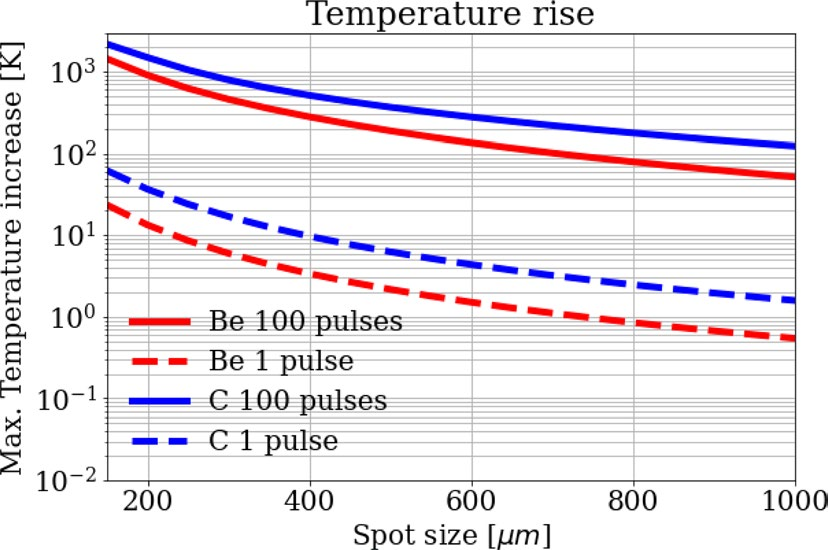
Maximum temperature rise after (dashed) a single pulse of 3 × 10^11^ positrons and (solid) a train of 100 pulses as a function of the spot size, shown for Beryllium and Carbon

As a first step, the FDTD algorithm has been preliminary applied to a target subjected to a 10 ps single positron pulse to check the heating and cooling dynamics in the target. The calculated temperature evolution in the beam center is shown in Fig. 8 for both Beryllium (a) and Pyrolytic Graphite (b) targets. Three distinct temporal ranges (time scales) can be clearly distinguished:

(i) for *t* < 10 ps, the heat is produced by the interaction between positrons and the target, and a fast local temperature increase with time can be observed.
(ii) from 10 ps to 10 μs, after the end of the heating process, the heat remains confined within the area of the beam spot interaction for O(µs) and has not yet diffused. During such a long quasi-adiabatic process, the temperature increase remains nearly constant approximately to Δ*T* = *q/ρc*_*p*_, where *q* is the heat density deposited (see Fig. 5), and *ρc*_*p*_ is the heat capacity for unit volume. Obviously, since the heat density *q* is proportional to 1/*a*^2^, the temperature rise exhibits the same law too, as shown by the curves in Figs. 8 and 9 (dotted lines). As an example, for the spot size *a* = 300 μm the maximum energy density deposition in the beam center for the Beryllium target is *q*_max_ = 20.8 J⋅cm^-^^3^ (see blue curve in Fig. 5a), the density *ρ* = 1850 kg⋅m^-^^3^, the heat capacity *c*_*p*_ = 1860 J⋅kg^-1^⋅K^-1^ (at 300 K), and the calculated maximum temperature rise (for 1 pulse) becomes *ΔT*_*1,Be*_ = *q*_max_*/ρc*_*p*_ = 6.0 K (blue curve in Fig. 8a). In the same conditions, for Pyrolytic Graphite the maximum energy density deposition in the beam center is *q*_max_ = 28.1 J⋅cm^-^^3^ (see blue curve in Fig. 5b),  *ρ *= 2250 kg⋅m^-^^3^, *c*_*p*_ = 706 J⋅kg^-1^⋅K^-1^, and the calculated maximum temperature rise becomes *ΔT*_*1,C*_ = 17.7 K (blue curve in Fig. 8b).
(iii) for *t* > 10 μs, the heat diffuses radially from the beam center to the colder zones of the disk. The characteristic time at which the heat diffusion process becomes relevant can be evaluated by
  6 $${t}_{c}=\frac{{a}^{2}}{4D\left({T}_{max}\right)}$$

where *D*(*T*_max_) = *k/ρc*_*p*_ is the thermal diffusivity corresponding to the target maximum temperature *T*_max_, i.e.,, the value reached in the second temporal range. As an example, for Beryllium at 300 K, assuming the spot size of *a* = 300 μm (Fig. 8a, blue curve), the diffusion time is *t*_*c*_ = 390 μs as calculated from Eq. 6, being the thermal conductivity *k* = 200 W⋅m^-1^K^-1^, and the thermal diffusivity *D* = 5.8 × 10^–5^ m^2^⋅s^-1^. On the other hand, for the Pyrolytic Graphite, in the same conditions, it results *t*_*c*_ = 18.4 μs (Fig. 8b, red curve), roughly 20 times smaller than for the Beryllium target, using *k*_*||*_= 1950 W⋅m^-1^K^-1^, and *D* = *k*_*||*_*/ρc*_*p*_ = 1.23 × 10^–3^ m^2^⋅s^-1^. It has to be noted that the diffusion process along the *z* axis is almost negligible here because there are no relevant temperature gradients along *z*. Moreover, also the *IR* emission from the target surface provides a weak heat evacuation process with a longer characteristic decay time, which cannot contribute to the decrease of the temperature at such short timescales. This becomes a relevant heat dissipation mechanism at the longer time scale of 10–100 s (depending on the target sizes), allowing the target to reach a steady temperature when subjected to the continuous sequence of bunches (see Fig. 2), as will be discussed later.

**Fig. 10:**
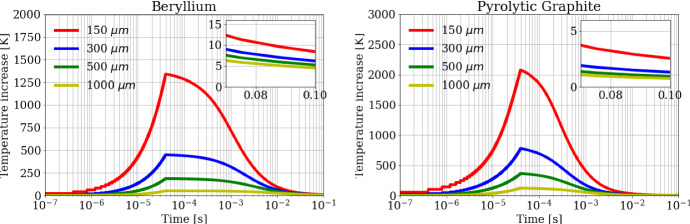
Temperature rise in the position corresponding to the beam center (*r* = 0), as a function of time, for a train of 100 bunches of 3 × 10^11^ positrons each impinging on the (left) Beryllium and (right) Carbon target for different beam spot sizes. The zoomed inserts highlight the *residual* temperature increase after 0.1 s from the start of the bunch train, which corresponds to the arrival of the subsequent train of bunches

As a second step of the analysis, we discuss the case of a train of *N* = 100 bunches of positrons, equally spaced in a time span of 40 μs, (see Fig. 2; Table1), crossing and heating consecutively the target every 400 ns. At such a timescale the *i*-th bunch hits the target when the heat deposited by the previous (*i* − 1)-th bunch has not yet diffused outside the beam spot. Therefore, the heat deposited by the whole train of *N* = 100 bunches is *piled-up* and the maximum temperature rise is reached in the beam center at the end of the heating process, at *t* = 40 μs. As an example, the calculated temperature rise in the beam center is shown for both Beryllium (Fig. 10a) and Pyrolytic Graphite (Fig. 10b) in the long timescale of 0.1 s. The four curves correspond to several beam spot sizes (*a* = 150  μm, 300  μm, 500  μm, 1000 μm). As expected the temperature peak is reached for all curves at the end of the train, after 40 μs. Looking at the specific case of *a* = 300 µm the maximum temperature rise (for 100 bunches) is *ΔT*_*N,Be*_ = 451 K for Beryllium (blue curve in Fig. 10a), and *ΔT*_*N,C*_ = 782 K for Pyrolytic Graphite (blue curve in Fig. 10b). By comparing these values with the analogous ones for the single pulse (*ΔT*_*1,Be*_ = 6.0 K and *ΔT*_*1,C*_ = 17.7 K), one may note that:

(i) *ΔT*_*N*_ < *N∙ΔT*_*1*_; the maximum temperature rise *ΔT*_*N*_ for *N* = 100 bunches is always lower than *N* = 100 times the maximum temperature rise for a single pulse *ΔT*_*1*_, as clearly shown also in Fig. 9. This happens for both Beryllium and Pyrolytic Graphite samples due to two cooperative effects: the specific heat increases with temperature causing the nonlinear behavior between temperature and heat density; the diffusion process provides a temperature peak reduction at a microsecond scale. This means that a small, but not negligible, fraction of heat starts diffusing before the end of the train. Such a cooling effect is more evident in Pyrolytic Graphite, being more diffusive than Beryllium;
(ii) although the maximum temperature rise (heat density) for Pyrolytic Graphite is around 70 % (40 %) larger than for Beryllium, however the melting point for Pyrolytic Graphite (~ 3900 K) is much higher than for Beryllium (~ 1550 K), making the choice of the Pyrolytic Graphite target potentially preferable for high power applications as in LEMMA.

As a third step, according to the full time structure of the positron pulsed beam described in Sect. 2.1, we studied the case of a series of bunch trains hitting the same point of the target with a cyclic repetition rate of 10 Hz (corresponding to the radiofrequency cavity length [8]). In this simulation we assumed that the energy deposition is the same for each pulse, neglecting any material physical/chemical property change or structural damage of the target, in a static and unconstrained configuration and discretizing the system as sketched in Fig. 7.

Given the long-time separation between two subsequent trains of bunches (*t*_rep_ = 0.1 s), each train hits the target when the heat generated by the previous one is almost completely diffused away from the beam spot. However, the thermal dissipation process is not fully completed and a small residual temperature rise remains at *t*_rep_ = 0.1 s, as the next train hits the sample, leading, in the long period, to a dangerous accumulation of thermal energy. As an example, the insets of Fig. 10 shows the zoom of the temperature rise around *t*_rep_ = 0.1 s, so to highlight the residual temperature increase in the target just before the arrival of the next train of bunches. Figure 10 shows how the residual temperature for Pyrolytic Graphite is about five times smaller than for Beryllium due to the larger thermal diffusivity.

**Fig. 11:**
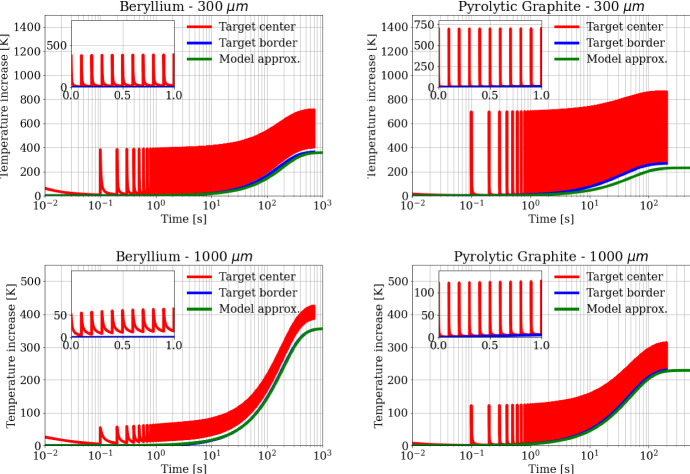
Temperature trends for (left) Beryllium and (right) Carbon targets following a train of 100 bunches at a train repetition rate of 10 Hz. Plots in the top row show the time evolution for a beam spot size of 300 μm while the bottom row for a beam spot size of 1000 μm. The red curves represent the temperature increase at the target center, the blue curves represent the temperature increase at the target border, and the green curves represent the model approximation for target average temperature

The numerical results are shown in Fig. 11 for a time interval of hundreds of seconds, when the target is approaching a steady state regime. The insets of Fig. 11 shows the temperature trend within 1 s, where it should be noted that already in such a time interval there is a thermal upward drift due to the residual temperature at the end of each *t*_rep_ period, as expected. Figure 10 shows the simulation results both for the target center and the target border (red and blue curves in the plots, respectively). It can be noticed how the pulsed beam structure is not affecting the evolution of the border temperature, which is not showing the multi-peak structure visible for the target center.

The larger increase in the temperature peak of Pyrolytic Graphite with respect to Beryllium is due to the higher heat density and lower heat capacity, as discussed before and shown for a train pulse in Fig. 10.

By solving the nonlinear ordinary differential equation in Eq. 4, one obtains the temperature increase for the target on timescales longer than the single train repetition rate, hence integrating the effect of multiple trains on the target over time. The emissivity *ɛ* appearing in Eq. 4 has been set to 0.8 for Beryllium and 0.98 for Pyrolytic Graphite. The result of this approximated model is overlaid in Fig. 11 (green curves) to the fine-grained temperature increase simulations. This approximated model is in agreement with the fine-grained one within 10 %. This agreement improves when the temperature in the target is more homogeneous, i.e., when the beam spot size is larger. This is indeed expected, since one of the assumptions in this approximation consists in considering only the average temperature of the target, neglecting any radial dependence of the temperature.

**Fig. 12:**
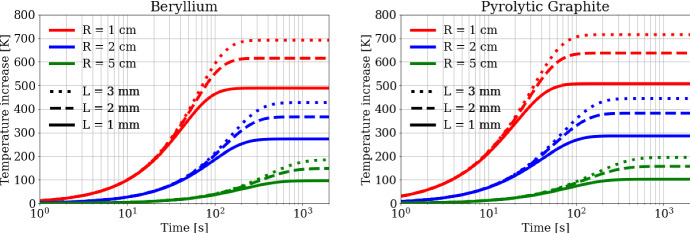
Temporal variation of residual temperatures and steady state temperatures, obtained with the simplified model, for (left) Beryllium and (right) Carbon

The results of the aforementioned approximated model are shown in Fig. 12 for different sizes of the considered Beryllium and Pyrolytic Graphite targets. The temperature evolution observed by solving Eq. 4 on this longer timescale is in agreement with an interpolation of the residual temperature increases obtained after each bunch, *t*_*rep*_, mentioned earlier in this section and visible in Fig. 11, within 10 %.

After a transient time ranging from 100  s to 1000 s, the targets reach the steady state temperature above room temperature (see Eq. 5). In particular, the targets reach the equilibrium temperature between 100 °C and 200 °C for thicknesses ranging between 1  mm and 3 mm in correspondence to *R* = 5 cm, between 250 °C and 450 °C in the case of *R* = 2 cm, and in the range 450–750 °C for *R* = 1 cm. As expected, Carbon (Pyrolytic Graphite) always reaches the equilibrium temperature on a shorter timescale as it has a lower specific heat than Beryllium. All these temperatures are well below the melting point of the considered material. The size and shape of the target can therefore be engineered with the aim of managing the optimum equilibrium temperature value for the target operation mode.

## Thermal Stress Analysis

Having introduced the approach to determine the space–time temperature field and compared the numerical results for Beryllium and Pyrolytic Graphite cylindrical targets of different sizes, in this section we describe the theoretical model used to determine the thermo-mechanical stresses induced in the target by the temperature gradients, which is eventually applied to the case study for a final comparison.

### Theoretical Model

In this paper we consider a solid target without constraints, i.e., free to expand. Both governing thermo-elastic equations and related boundary conditions are expressed in terms of thermal stresses. In this case the stress compatibility equation is of the Beltrami-Michell type, where the body forces are neglected [50]. Since the deposited heat density, as well as the thermal field distribution, has a negligible dependence on the axial coordinate *z* (thin target), the problem can be approximated to the case of axially unrestrained plane strain, assuming the latter to be constant [51–54].

In cylindrical coordinates with angular symmetry (i.e., $\frac{\partial }{\partial \phi }=0$) normal strains are

7 $${\varepsilon }_{rr}=\frac{{\partial u}_{r}}{\partial r};\, {\varepsilon }_{\phi \phi }=\frac{{u}_{r}}{r};\, {\varepsilon }_{zz}=\frac{{\partial u}_{z}}{\partial z}$$

where *u*_*r*_ and *u*_*z*_ are the radial and axial displacement, respectively.

The condition based on which the elastic deformation preserves the planarity of the disk cross section (i.e., the axial deformation does not depend on the target radius) defines the case of *generalized plane strain*, which is mathematically expressed by $\frac{\partial {\varepsilon }_{zz}}{\partial r}=0$. The stress–strain relations in terms of Young’s modulus and Poisson’s ratio are

8 $$\left\{\begin{array}{c}{\varepsilon }_{rr}=\frac{1}{E}\left[{\sigma }_{rr}-\nu \left({\sigma }_{\phi \phi }+{\sigma }_{zz}\right)\right]+\alpha \Delta T(r)\\ {\varepsilon }_{\phi \phi }=\frac{1}{E}\left[{\sigma }_{\phi \phi }-\nu \left({\sigma }_{zz}+{\sigma }_{rr}\right)\right]+\alpha \Delta T(r)\\ {\varepsilon }_{zz}=\frac{1}{E}\left[{\sigma }_{zz}-\nu \left({\sigma }_{rr}+{\sigma }_{\phi \phi }\right)\right]+\alpha \Delta T(r)\end{array}\right.$$

where *σ*_*rr*_, *σ*_*ϕϕ*_ and *σ*_*zz*_ are the radial, hoop and axial stresses, *E* is Young's modulus, *α* the thermal expansion coefficient, *ν* is the Poisson's ratio and *ΔT(r)* = *T(r) − T*_*0*_ is the radial temperature variation. A constant axial deformation (*ε*_*zz*_ = *ε*_*0*_) maintains the body in the plane deformation state following the axial stress σ_zz_ acting on the cross sections of the disk. The axial stress σ_zz_ depends on the normal stresses σ_rr_ and σ_ϕϕ_. The constant axial strain *ε*_*0*_ for a plain strain can be determined from the condition that the axial force is zero.

9 $$2\pi {\int }_{0}^{R}{\sigma }_{zz}\cdot r\cdot dr=0$$

Solving Eq. 9 for stresses in terms of strains yields to

10 $$\left\{ {\begin{array}{*{20}c} {\sigma _{{rr}} = } & {\frac{E}{{\left( {1 + \nu } \right)\left( {1 - 2\nu } \right)}}\left[ {\left( {1 - \nu } \right)\varepsilon _{{rr}} + \nu \varepsilon _{{\phi \phi }} - \left( {1 + \nu } \right)\alpha \Delta T} \right]} \\ {\sigma _{{\phi \phi }} = } & {\frac{E}{{\left( {1 + \nu } \right)\left( {1 - 2\nu } \right)}}\left[ {\left( {1 - \nu } \right)\varepsilon _{{\phi \phi }} + \nu \varepsilon _{{rr}} - \left( {1 + \nu } \right)\alpha \Delta T} \right]} \\ {\sigma _{{zz}} = } & {\nu \left( {\sigma _{{rr}} + \sigma _{{\phi \phi }} } \right) - E\alpha \Delta T} \\ \end{array} } \right.$$

and the equilibrium equation for axial symmetry is.

11 $$\frac{d{\sigma }_{rr}}{dr}+\frac{{\sigma }_{rr}-{\sigma }_{\phi \phi }}{r}=0$$

By following this analytical approach and taking into account the conditions in Eq. 8, we obtain the following expressions for stresses:

12 $$\left\{ {\begin{array}{*{20}c} {\sigma _{{rr}} = } & {\frac{E}{{1 - \nu }}\left[ {\frac{1}{{R^{2} }}\smallint _{0}^{R} \alpha \Delta T\left( {r,t} \right) \cdot r \cdot dr - \frac{1}{{r^{2} }}\smallint _{0}^{r} \alpha \Delta T\left( {r,t} \right) \cdot r \cdot dr} \right]} \\ {\sigma _{{\phi \phi }} = } & {\frac{E}{{1 - \nu }}\left[ {\frac{1}{{R^{2} }}\smallint _{0}^{R} \alpha \Delta T\left( {r,t} \right) \cdot r \cdot dr + \frac{1}{{r^{2} }}\smallint _{0}^{r} \alpha \Delta T\left( {r,t} \right) \cdot r \cdot dr - \alpha \Delta T\left( {r,t} \right)} \right]} \\ {\sigma _{{zz}} = } & {\frac{E}{{1 - \nu }}\left[ {\frac{2}{{R^{2} }}\smallint _{0}^{R} \alpha \Delta T\left( {r,t} \right) \cdot r \cdot dr - \alpha \Delta T\left( {r,t} \right)} \right]} \\ \end{array} } \right.$$

### Stability Analysis and Safety Factor

In the analysis of the potential yielding and fracture of the targets, the Christensen generalized failure criterion has been chosen [55], which has general validity, being more inclusive and suitable for both materials with brittle failure characteristics and for those having ductile behaviors and plastic yielding. In the considered case, assuming the axisymmetric configuration and the generalized plane strain condition, it can be expressed as follows:

13 $$\left(\frac{1}{{\sigma }_{T}}-\frac{1}{{\sigma }_{C}}\right)\left({\sigma }_{rr}+{\sigma }_{\phi \phi }+{\sigma }_{zz}\right)+\frac{1}{2{\sigma }_{T}{\sigma }_{C}}\left[{\left({\sigma }_{rr}-{\sigma }_{\phi \phi }\right)}^{2}+{\left({\sigma }_{\phi \phi }-{\sigma }_{zz}\right)}^{2}+{\left({\sigma }_{zz}-{\sigma }_{rr}\right)}^{2}\right]\le 1$$

where *σ*_*T*_ and *σ*_*C*_ are the tensile and compressive strength, respectively.

### Numerical Simulations and Discussion

**Fig. 13:**
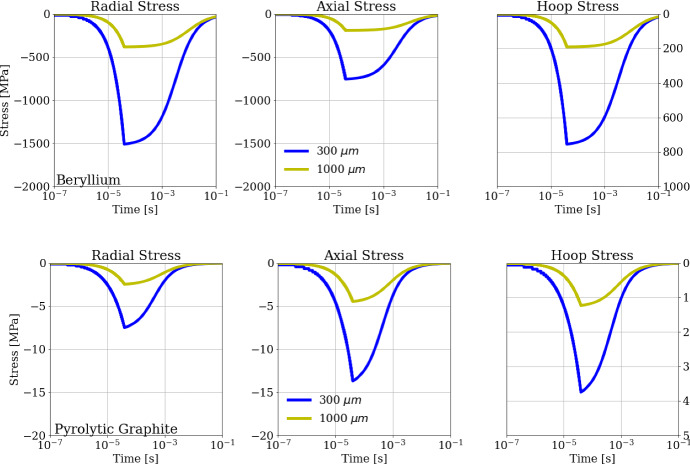
Radial, hoop and axial stresses for (top row) Beryllium and (bottom row) Pyrolytic Graphite targets, for different beam spot sizes

In this section we analyze the thermomechanical stresses induced by the positron sources described in the case study. The radial, axial, and hoop stresses are calculated by applying Eq. 12 to the space–time thermal field evaluated in Sect. 3. The largest values of the stress are achieved during the strong heating process in the first 40 μs, when fast relevant thermal gradients occur. For this reason, we limit the thermo-mechanical stress analysis to the first cycle only, as shown in Fig. 13 for both target materials. In the case of a beam size *a* = 300 μm, the radial stress reaches a peak absolute value of about 1500 MPa for the Beryllium target, being instead only 7.5 MPa for the Pyrolytic Graphite. Note that for both materials the radial and axial stresses have a negative sign due to the compressive nature of the thermo-mechanical stress. This is particularly important because brittle materials, unlike ductile ones, have a higher compressive strength than tensile strength [56, 57].

**Fig. 14:**
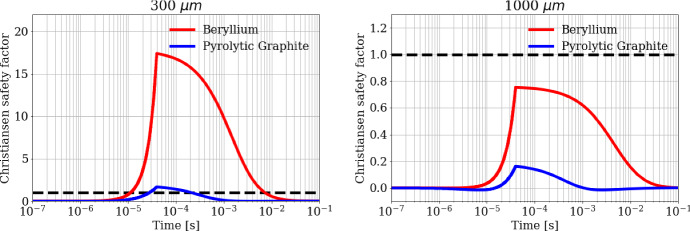
Christiansen safety factor evolution during time for a beam spot size of (left) 300 μm and (right) 1000 μm, for Beryllium and Pyrolytic Graphite. The horizontal dashed line corresponds to the safety threshold of 1.0

The evaluation of the stress with respect to the Christiansen safety condition is illustrated in Fig. 14, where the Christiansen quality factor, well represented by the left-side of Eq. 13, is reported as a function of time, for a train of bunches crossing a Beryllium or Pyrolytic Graphite target with beam sizes of 300 μm and 1000 μm respectively. The dotted black line, kept fixed at 1, represents the limit value not to be overpassed to avoid the target failure. In the evaluation of Eq. 13, we have used the tensile and compressive strengths as a function of temperature: for Beryllium in the range 300–1100 K the quantity *σ*_*T*_ decreases with temperature from 550  MPa to 80 MPa, while *σ*_*C*_ decreases from 600  MPa to 90 MPa. For Pyrolytic Graphite in the range 300–1100 K (due to the mechanical resistance increase with temperature) the quantity *σ*_*T*_ increases from 7.5  MPa to 12.5 MPa, while *σ*_*C*_ increases from 9.5  MPa to 15.5 MPa.

It is worth noting that for the case of *a* = 300 μm, the adopted Christiansen criterion is unfulfilled after O(10^–5^ s) for both Beryllium and Pyrolytic Graphite, while in the case of *a* = 1000 μm the criterion is satisfied for both the considered materials. The Christensen safety factor is significantly higher for Beryllium than for Pyrolytic Graphite: it is 10 times larger in case of *a* = 300 μm, while it becomes 5 times for *a* = 1000 µm. It was reasonable to expect similar results since the Thermal Shock Resistance Parameter *R*″ = *Dσ*_*C*_*/αE* [m^2^K⋅s^-1^] is higher for Pyrolytic Graphite than for Beryllium [58–63]. In particular, *R*″ assumes values two orders of magnitude higher for Pyrolytic Graphite than for Beryllium in the temperature range investigated. The PEDD (Peak Energy Deposition Density) values, relative to the 1000 µm spot, for the assumed bunch sequence is 101.3 J⋅g^-1^ and 112.3 J⋅g^-1^ for Beryllium and Carbon (Pyrolytic Graphite), respectively [64–68].

## Conclusions and Perspectives

The design of a muon collider as a future accelerator to search for new types of elementary interactions requires the study of advanced technologies in material science. For a positron-driven muon collider (LEMMA) one key element is the study of material properties in presence of a peculiar pattern of energy deposition in time and space due to the continuous ionization and the consequent thermal load.

In this paper we have developed a theoretical model to simulate the temperature increase of a target subjected to very fast sequences of positron pulses, over different timescales, from ps regime to hundreds of seconds. Moreover we have provided a simple method to estimate the induced thermomechanical stresses in the target, together with Christensen safety factor criteria to prevent the crack formation mechanism.

Such a theoretical approach has been applied to a specific case study where cylindrical targets of Beryllium or Pyrolytic Graphite are subjected to fast trains of 100 bunches of intense positron beams (3 × 10^11^ positrons/bunch). Each train has a time duration of 40 μs and is repeated cyclically every 0.1 s. The numerical results for a Gaussian beam spot size with *a* = 300 μm show a fast temperature increase during the heating cycle of Δ*T* = 451 K for Beryllium and Δ*T*  = 782 K for Pyrolytic Graphite, corresponding to a maximum radial stress of about 1500 MPa for Beryllium and 7.5 MPa for Pyrolytic Graphite, neither one therefore satisfying the Christensen safety factor. A possible way to make the thermomechanical stresses sustainable is to use a less-focused positron beam. As an example, with a spot size of 1000 μm the maximum radial stress drops to 400 MPa for Beryllium and 2 MPa for Pyrolytic Graphite.

Moreover, we demonstrated that in a longer timescale (~ 100 s) the target tends to reach a steady state temperature *T*_SS_ that can be estimated from the energy balance between the heat deposition from the pulsed source and the thermal radiation loss with the environment. A useful simplified formula has been introduced here to estimate *T*_SS_ (with an uncertainty of ~ 10 %), showing a clear relationship with both the emissivity and the geometrical shape and sizes of the target, whose design could be optimised.

For all the reasons described above, the comparison between Beryllium and Pyrolytic Graphite has highlighted how Graphite exhibits better thermal properties and safety margins in terms of maximum temperature increase, heat diffusion, infrared emissivity, and resistance to thermo-mechanical stresses, thus resulting a better candidate material for future developments of muon colliders, where an efficient muon production will come always with a high thermal energy deposition on the target.

We have developed a flexible tool to study the target thermal field and the thermo-mechanical stress, which can be applied more in general to any material, target geometry, sequences and intensities of the positron bunches. In principle, our model can be also easily extended to other particle sources, as protons or electrons, for which the heat deposition can be again calculated with FLUKA and Geant4 [69–71].

It would be desirable to develop a suitable experimental setup to measure the surface thermal field induced by high energy electron or positron beams through accurate diagnostic techniques, thus testing part of the conclusions reached in this article.

A complementary theoretical study should be devoted in the future to investigate new materials [72–81], and functionalized multi-layered targets with better performances, keeping the thermo-mechanical stability as the beam spot size is reduced and the intensity of the source increased, that is the current challenge in the muon collider.

## Figures and Tables

**Fig. 15:**
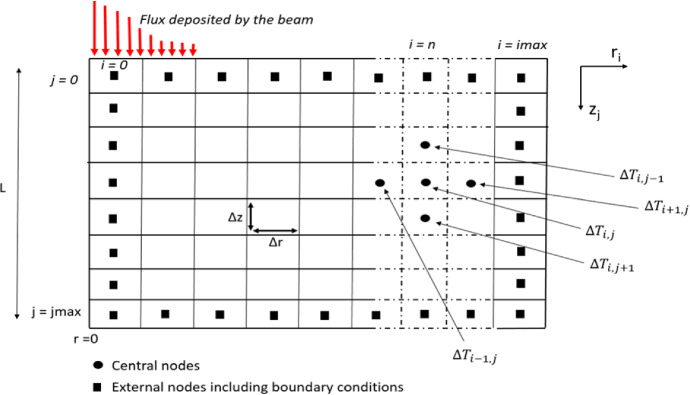
Schematic representation of numerical model implementation for temperature variation inside the material. The target volume is divided in [Δ*r*, Δ*z*] cells where every cell is a node in the computation. The incoming beam is approaching the target along the *z* axis from the top

## A. Material Properties

We report explicitly the temperature dependence of the material thermoelastic parameters that we have used in the FDTD algorithm for the evaluation of the target space–time thermal field, and thermomechanical stresses. Tables

**Table 2:** Thermal parameters of Beryllium and Carbon (Pyrolytic Graphite) as a function of the temperature, expressed in Kelvin, valid in the range 300–1500 K

Be	Thermal conductivity [W⋅mK]	$k=430.35-1.1674T+1.6044\cdot {10}^{-3}{T}^{2}-1.0097\cdot {10}^{-6}{T}^{3}+2.3642\cdot {10}^{-10}{T}^{4}$
	Specific heat [J⋅KgK]	${c}_{p}=606.91+5.3382T-4.1726\cdot {10}^{-3}{T}^{2}+1.2723\cdot {10}^{-6}{T}^{3}$
C	Thermal conductivity [W⋅mK]	*k*_*//*_$=-1.72\cdot {10}^{2}+6.26\cdot {10}^{5}/T-1.06\cdot {10}^{5}/{T}^{2}-6.62\cdot {10}^{-2} T$ *k*_*⊥*_$=-3.79\cdot {10}^{-1} +1.804\cdot {10}^{3}/T-8.23\cdot {10}^{2}/{T}^{2}+0.17\cdot {10}^{-3}T$
	Specific heat [J⋅KgK]	${c}_{p}=-474.0+4.9532T-3.6093\cdot {10}^{-3}{T}^{2}+9.3068\cdot {10}^{-7}{T}^{3}$

^2^ and

**Table 3:** Structural parameters of Beryllium and Carbon (Pyrolytic Graphite) as a function of temperature, expressed in Kelvin, valid in the range 300–1500 K

Be	Young modulus [GPa]	$E=297{e}^{-3.5p}\left[1-1.9\cdot {10}^{-4}\left(T-293\right)\right]$(p is the volume fraction of pores)
	Thermal expansion coeff. [10^−6^ K^−1^]	$\alpha =8.4305+1.1464\cdot {10}^{-2}T-2.9752\cdot {10}^{-6}{T}^{2}$
C	Young modulus [GPa]	$E=5.24+3.1429\cdot {10}^{-2}T+5.4286\cdot {10}^{-7}{T}^{2}$
	Thermal expansion coeff. [10^−6^ K^−1^]	${\alpha }_{a-dir}=1.6\cdot {10}^{-6}\left[1+{\left(\frac{\Delta \alpha }{\alpha }\right)}_{a-{\text{dir}}}\right]$ ${\text{where}} {\left(\frac{\Delta \alpha }{\alpha }\right)}_{a-dir}=\left(-9.3391\cdot {10}^{-2}+2.9624{\cdot 10}^{-4}T+9.0036\cdot {10}^{-8}{T}^{2}\right)\cdot {10}^{-2}$ ${\alpha }_{c-{\text{dir}}}=5.8\cdot {10}^{-6}\left[1+{\left(\frac{\Delta \alpha }{\alpha }\right)}_{c-dir}\right]$ ${\text{where}} {\left(\frac{\Delta \alpha }{\alpha }\right)}_{c-dir}=\left[2.7174{\cdot 10}^{-3}\left(T-300\right)\right]\cdot {10}^{-2}$

^3^ explicitly contain the temperature law of all the parameters used for Figs. 2 and 3.

The densities of Beryllium and Carbon (Pyrolytic Graphite) are 1850 kg⋅m^-^^3^ and 2250 kg⋅m^-^^3^, respectively.

## B. Numerical Model Implementation

We report in this appendix the technical details concerning the numerical model implemented for the determination of the temperature evolution in the target subjected to cyclic trains of positron beams. Thanks to the cylindrical shape of both target and positron beam (see Fig. 7), the temperature has also a cylindrical symmetry, and the FDTD algorithm has been implemented in a 2D space, i.e., in the *r,z* domain. The volume is discretized in a number of small cylindrical shells (voxels) of volume *ΔV* = *2*π*r*Δ*r*Δ*z*, corresponding to the 2π revolution of the area Δ*r∙*Δ*z* in the 2D space (see Fig. 15). Each voxel is identified by the index *i* = [0; *i*max] corresponding to the radial distance from the center *r*_*i*_ = (*i* + 0.5)*∙* Δ*r,* and by the index *j* = [0; *j*max] corresponding to the depth *z*_*j*_ = (*j* + *0.5*)*∙* Δ*z.* In each voxel the temperature increase Δ*T*_*i,j*_ and the thermal parameters are homogenous. The heat exchange among neighboring voxels can be calculated by discretizing Eq. 2, allowing to update the temperature increase of each voxel every Δ*t* as follows

B1 $${\Delta T}_{i,j}^{\text{new}}={\Delta T}_{i,j}^{old}+\left(\frac{{\Phi }_{i,j}^{\text{old}}}{{\Delta V}_{i,j}}+{\mathrm{w}}_{i,j}^{\text{old}}\right)\frac{\Delta t}{\rho {c}_{p i,j}}$$

where ${w}_{i,j}^{\text{old}}$ and ${\Phi }_{i,j}^{\text{old}}$ are heat density deposited per unit time and the incoming heat flux in the voxel. The superscript “*old*” and “*new*” stand for time *t* and *t* + Δ*t* respectively. For central nodes of the grid (symbol ● in Fig.15) the incoming flux ${\Phi }_{i,j}^{\text{old}}$ is given by the sum of the fluxes coming from the four neighbor cells as follows

B2 $${\Phi }_{i,j}^{\text{old}}={\Phi }_{i,j}^{\text{left}}+{\Phi }_{i,j}^{\text{right}}+{\Phi }_{i,j}^{\text{top}}+{\Phi }_{i,j}^{\text{bottom}}$$

being

B3 $$\left\{\begin{array}{c}{\Phi }_{i,j}^{\text{left}}=-{k}_{i,j}{S}_{\text{left}}\left(\frac{{\Delta T}_{i,j}^{\text{old}}-{\Delta T}_{i-1,j}^{\text{old}}}{\Delta r}\right) \\ {\Phi }_{i,j}^{\text{right}}=+ {k}_{i,j}{S}_{\text{right}}\left(\frac{{\Delta T}_{i+1,j}^{\text{old}}-{\Delta T}_{i,j}^{\text{old}}}{\Delta r}\right) \\ {\Phi }_{i,j}^{\text{top}}=-{k}_{i,j}{S}_{\text{top}}\left(\frac{{\Delta T}_{i,j}^{\text{old}}-{\Delta T}_{i,j-1}^{\text{old}}}{\Delta z}\right) \\ {\Phi }_{i,j}^{bottom}=+ {k}_{i,j}{S}_{\text{bottom}}\left(\frac{{\Delta T}_{i,j+1}^{\text{old}}-{\Delta T}_{i,j}^{\text{old}}}{\Delta z}\right)\end{array}\right.$$

where the interfaces between two neighbor voxels are

B4 $$\left\{ {\begin{array}{*{20}c} {S_{{{\text{left}}}} = } & {2\pi \left( {r_{i} - \frac{{\Delta r}}{2}} \right)\Delta z} \\ {S_{{{\text{right}}}} = } & {2\pi \left( {r_{i} + \frac{{\Delta r}}{2}} \right)\Delta z} \\ {S_{{{\text{top}}}} = } & {\pi \left[ {\left( {r_{i} + \frac{{\Delta r}}{2}} \right)^{2} - \left( {r_{i} - \frac{{\Delta r}}{2}} \right)^{2} } \right]} \\ {S_{{{\text{bottom}}}} = } & {S_{{{\text{top}}}} } \\ \end{array} } \right.$$

On the contrary the external nodes of the grid (symbol ■ in Fig.15) have always at least one interface with the external space kept at room temperature T_room_ (i.e., the vacuum chamber), and some of the heat fluxes shown in Eq.B3 should be replaced by the following outgoing heat fluxes via infrared radiation

B5 $$\left\{ {\begin{array}{*{20}c} {\Phi _{{0,j}}^{{{\text{left}}}} = } & 0 \\ {\Phi _{{i{\text{max}},j}}^{{{\text{right}}}} = } & { - \varepsilon \sigma S_{{{\text{right}}}} \left( {\left( {\Delta T_{{i{\text{max}},j}}^{{old}} + T_{0} } \right)^{4} - T_{{{\text{room}}}}^{4} } \right)} \\ {\Phi _{{i,0}}^{{top}} = } & { - \varepsilon \sigma S_{{{\text{top}}}} \left( {\left( {\Delta T_{{i,0}}^{{{\text{old}}}} + T_{0} } \right)^{4} - T_{{{\text{room}}}}^{4} } \right)} \\ {\Phi _{{i,j{\text{max}}}}^{{{\text{bottom}}}} = } & { - \varepsilon \sigma S_{{{\text{bottom}}}} \left( {\left( {\Delta T_{{i,j{\text{max}}}}^{{old}} + T_{0} } \right)^{4} - T_{{{\text{room}}}}^{4} } \right)} \\ \end{array} } \right.$$

where *ɛ* is the target emissivity, *σ* is the Stefan-Boltzmann constant, *T*_*0*_ is the initial temperature of the target. An exception is for the voxels located at the beam center (*i* = 0) for which ${\Phi }_{0,j}^{\text{left}}=0,$ due to the radial symmetry. Eq. B1 is eventually applied on each voxel iteratively, by choosing the time interval $\Delta t<\frac{{\text{min}}\left({\Delta r}^{2},{\Delta z}^{2}\right)}{2{D}_{\text{max}}}$ to fulfill the stability condition, and to guarantee the convergence of the solution at all the timescales.

## Footnotes

1 Other processes, including the production of muon pairs from positron collision with a material electron, which is the process of interest for muon beam production, are largely subdominant, and not contributing significantly to the deposited energy in the target.

2 We describe the geometry of the target using cylindrical coordinates where *z* is the coordinate along the target axis, *r* is the radial position, and *ϕ* is the azimuthal angle.

3 The thicknesses considered correspond to 0.85 % X_0_ and 0.52 % X_0_ for Beryllium and Carbon, respectively, where X_0_ indicates the material radiation length [36].

4 The convection is neglected in this model assuming that the target is in vacuum as it must be for the positron and muon propagation.
